# Supplementary material for: Regular physical activity affects brain activities in old individuals: an observational study
Source: PLoS One. 2025 Jul 2;20(7):e0326163. doi: 10.1371/journal.pone.0326163 (PMC12220997; doi:10.1371/journal.pone.0326163)
Supplement: S1 Table — MMSE-J, Japanese version of Mini-Mental State Examination; FAB-J, Japanese version of Frontal Assessment Battery; ADAS-J cog, Japanese version of Alzheimer’s Disease Assessment Scale-Cognitive section; MF, Median Frequency; IAF, Individual Alpha Frequency; SSE, Shannon’s Spectral Entropy; r, Pearson’s correlation coefficient; p (FDR), p-value corrected for false discovery rate. (PDF) [file pone.0326163.s001.pdf]

S1 Table. Results of Correlation Analysis: Active Group

	Age		MMSE-J		FAB-J		ADAS-J cog		MF		IAF	
	r	*p* (FDR)	*r*	*p* (FDR)	*r*	*p* (FDR)	*r*	*p* (FDR)	*r*	*p* (FDR)	*r*	*p* (FDR)
MMSE-J	-0.102	0.261										
FAB-J	-0.200	0.026*	0.515	< 0.001*								
ADAS-J cog	0.210	0.026*	-0.858	< 0.001*	-0.411	< 0.001*						
MF	-0.010	0.902	0.281	0.001*	0.208	0.021*	-0.281	0.003*				
IAF	-0.101	0.261	0.349	< 0.001*	0.299	0.001*	-0.328	< 0.001*	0.670	< 0.001*		
SSE	0.027	0.781	0.108	0.256	0.057	0.548	-0.146	0.142	0.764	< 0.001*	0.316	< 0.001*

MMSE-J, Japanese version of Mini-Mental State Examination; FAB-J, Japanese version of Frontal Assessment Battery; ADAS-J cog, Japanese version of Alzheimer's Disease Assessment Scale-Cognitive section; MF, Median Frequency; IAF, Individual Alpha Frequency; SSE, Shannon's Spectral Entropy; r, Pearson's correlation coefficient; p (FDR), p-value corrected for false discovery rate.
