# Supplementary material for: Regular physical activity affects brain activities in old individuals: an observational study
Source: PLoS One. 2025 Jul 2;20(7):e0326163. doi: 10.1371/journal.pone.0326163 (PMC12220997; doi:10.1371/journal.pone.0326163)
Supplement: S2 Table — MMSE-J, Japanese version of Mini-Mental State Examination; FAB-J, Japanese version of Frontal Assessment Battery; ADAS-J cog, Japanese version of Alzheimer’s Disease Assessment Scale-Cognitive section; MF, Median Frequency; IAF, Individual Alpha Frequency; SSE, Shannon’s Spectral Entropy; r, Pearson’s correlation coefficient; p (FDR), p-value corrected for false discovery rate. (PDF) [file pone.0326163.s002.pdf]

S2 Table. Results of Correlation Analysis: Nonactive Group

	Age		MMSE-J		FAB-J		ADAS-J cog		MF		IAF	
	r	*p* (FDR)	*r*	*p* (FDR)	*r*	*p* (FDR)	*r*	*p* (FDR)	*r*	*p* (FDR)	*r*	*p* (FDR)
MMSE-J	-0.257	0.001*										
FAB-J	-0.200	0.010*	0.637	< 0.001*								
ADAS-J cog	0.274	0.002*	-0.808	< 0.001*	-0.639	< 0.001*						
MF	-0.084	0.274	0.396	< 0.001*	0.405	< 0.001*	-0.398	< 0.001*				
IAF	-0.065	0.390	0.386	< 0.001*	0.375	< 0.001*	-0.377	< 0.001*	0.847	< 0.001*		
SSE	-0.087	0.274	0.168	0.030*	0.251	0.001*	-0.187	0.034*	0.756	< 0.001*	0.510	< 0.001*

MMSE-J, Japanese version of Mini-Mental State Examination; FAB-J, Japanese version of Frontal Assessment Battery; ADAS-J cog, Japanese version of Alzheimer's Disease Assessment Scale-Cognitive section; MF, Median Frequency; IAF, Individual Alpha Frequency; SSE, Shannon's Spectral Entropy; r, Pearson's correlation coefficient; p (FDR), p-value corrected for false discovery rate.
