# Supplementary material for: Regular physical activity affects brain activities in old individuals: an observational study
Source: PLoS One. 2025 Jul 2;20(7):e0326163. doi: 10.1371/journal.pone.0326163 (PMC12220997; doi:10.1371/journal.pone.0326163)
Supplement: Attachment — Submitted filename: Response to Reviewers.docx [file pone.0326163.s004.docx]

Response to reviewers:

Regular physical activity affects brain activities in old individuals:

an observational study

Short title: Physical activity and brain activity

Keisuke Fukasawa^1, ¶^, Hideyuki Hoshi^2,3, ¶^, Yoko Hirata^4^, Momoko Kobayashi^3^, Keita Shibamiya^3^, Sayuri Ichikawa^1^, Yoshihito Shigihara^2,3*^

^1^Clinical Laboratory, Kumagaya General Hospital, Kumagaya City, Saitama, Japan,

^2^Precision Medicine Centre, Hokuto Hospital, 7-5 Kisen, Inada-cho, Obihiro-shi, Hokkaido, Japan

^3^Precision Medicine Centre, Kumagaya General Hospital, Kumagaya City, Saitama, Japan

^4^Department of Neurosurgery, Kumagaya General Hospital, Kumagaya City, Saitama, Japan

* Corresponding author Yoshihito Shigihara

E-mail: [y-shigihara@hokuto7.or.jp](mailto:y-shigihara@hokuto7.or.jp) (YS)

^¶^These authors contributed equally to this work.

To the editor and reviewers:

Thank you for giving us the opportunity to submit a revised draft of our manuscript titled ‘Regular physical activity affects brain activities in old individuals: an observational study’.

We appreciate the time and effort you have dedicated to providing valuable feedback on our manuscript. We are grateful for your insightful comments that helped us improve our paper's quality. We have incorporated changes to reflect all of your suggestions.

Please find our point-by-point responses to all comments and concerns below.

Role of Funder statement

This study was partially supported by RICOH Co., Ltd. The funders had no role in study design, data collection and analysis, decision to publish, or preparation of the manuscript.

Response to Reviewer 1 Comments

Comment 1-1:

The design of the study is not prospective, just retrospective transverse research. Therefore, there is a problem suggesting the reverse results; the high cognitive function people tend to perform more physical activities. Furthermore, this study was done in one institute, then the bias of the subjects could not be avoided. One suggested plan is that the repeated research on different subjects might be more stronger influences on this result.

Response:

Thank you for your comments and suggestions. Following the comment, we have modified the manuscript in lines 434-437 and 437-450 in the revised manuscript.

Regarding the first point, we fully agree that there is a possibility of reverse results. As mentioned, it is an unavoidable limitation of the present study because it was an observation study. Although we had already mentioned this in the limitation section of the original manuscript, we have further clarified it (pages 26-27, lines 437-450). While the possibility of reverse causality remains, this study provides meaningful insight for the clinical practice. The MEG spectral parameters were different between groups (‘Active’ vs. ‘Nonactive’), indicating that the parameters are good indicators for physicians to infer patients’ lifestyles. We believe that it does not matter if the neurological differences yielded the patients’ active/nonactive behaviour or whether the patients’ active/nonactive lifestyles yielded the neurological differences, because the results could still contribute to properly assessing patients’ conditions and designing effective therapeutic strategies.

Regarding the second point, the claim is on point. We have acknowledged that this is one of the limitations of the present study (page 27, lines 434-437). To address the concern, we are currently preparing to replicate the study in another hospital equipped with MEG. Furthermore, we know that another MEG site in Japan is planning similar studies. We hope that future studies from those MEG sites would provide supportive evidence.

Comment 1-2:

Confounding factors such as diet, social activities, educational level may affect the cognitive functions.

Response:

Thank you for the insightful comment. Accordingly, we have added descriptions regarding the relationships between regular physical activity and other lifestyle-associated (‘confounding’) factors (pages 23-24, lines 370-384) and added a limitation regarding the confounds (pages 27-28, lines 450-458).

Lifestyle-associated factors interact with each other. They synergically modify the cognitive state. For example, a regular exercise routine contributes to a reduced risk of cognitive decline both directly and indirectly. The indirect pathway manifests via changing other risk factors (e.g., reducing the risk of hypertension). From a clinical perspective, it is not important to discuss how much each lifestyle-associated factor directly contributed to (reducing) the risk of cognitive decline. Therefore, we considered the effects of a regular exercise routine as a whole, which includes their interactive effects with other lifestyle-associated factors (i.e., indirect effects), and focused on the comprehensive effect of regular exercise routines, which is an interest in clinical practice.

However, some lifestyle-associated factors or even other factors could influence MEG spectral parameters independently from regular physical activity. We cannot exclude this possibility and cannot address these factors because this was a retrospective observational study: we do not have enough information about them. We described this as the third limitation (pages 27-28, lines 450-458).

Comment 1-3:

In my experience, the relationship between MEG results and cognitive function is considered to be difficult to understand.

Response:

Thank you for your comment. To address these doubts, we have further elaborated on the use of MEG as an assessment of cognitive decline (pages 5-6, lines 91-94 and page 14, lines 237-239).

We respect your view and understand that the majority of physicians have the impression that the use of MEG (and EEG, electrophysiological examinations in general) is difficult, except for patients with epilepsy. However, the comprehensibility of MEG results depends on the environment and strategies of clinical practice teams. We have been using MEG in the clinical practice for assessing patients with cognitive decline for 5 years. This has been enabled by the development of an analysis pipeline that reduces the analysis time/effort for technicians, by providing the quantification of the MEG measurements (e.g., MF, IAF, SSE, and others), and by summarising the results using a comprehensible format for all hospital staff member. We have been using this approach because we often receive feedback from hospital staff (i.e., physicians, clinical psychologists, clinical laboratory technicians, and therapists) that the results are clinically useful. Changes in MEG spectral parameters (i.e., MF, IAF, and SSE) are well matched with our clinical impressions [1]. We always show MEG data in two dimensional scatter plots along with patient data [2] or show their trends using line charts [1]. These are powerful tools to encourage patients and their families to change their lifestyle. Details of our clinical experiences have been published as a case series [1].

Comment 1-4:

Data availability is not clearly written. DOI is not effective.

Response:

We apologise the inconvenience. Unfortunately, we cannot reproduce the inaccessibility of the dataset using the doi: 10.17632/xgzgpk9475.1 (https://doi.org/10.17632/xgzgpk9475.1). Two of authors resolved the doi and verified that the link is working using independent environments. Could you please retry and let us know if it works?

Response to Reviewer 2 Comments

Comment 2-1

Can the authors provide references to explanations of values MF, IAF, SSE (l. 84).

Response:

Thank you for your question. According to this comment and comments by other reviewers, we have added a new subsection to describe the details of the spectral parameters (‘MEG analysis’ section; pages 12-14, lines 201-239). The pipelines for computing the MF, IAF, and SSE values are also extensively explained with references in this subsection.

Comment 2-2

Abbreviations in main text differ from those in the abstract (34-38) For example, in the abstract you note MMSE-J, but in the text just MMSE (53).

Response:

Thank you for your detailed reading and the note. Following the comment, we have carefully checked the wording throughout the manuscript. We intentionally used these two abbreviations with different meanings. We use ‘MMSE’ to refer to the Mini-Metal State Examination (in general) including original and translated versions, whereas we use ‘MMSE-J’ to specifically refer to its Japanese version which we used in the study. The validity and equivalence to the original version has already been evaluated [3]. Additionally, the copyright holders of the different versions are different; thus, we intestinally distinguished them.

Comment 2-3:

7, 111 – give corresponding statistics for men, especially statistics of age.

Response:

Thank you for the comment. The statistics (mean age and SD), which were described in the original manuscript, were not for women alone but included all patients. We apologies for the confusion. The number of men had been omitted in the original the manuscript, because we thought it would be inherently clear (total participants minus the number of women). Following the comment, we have added the number of the men (N = 139) (page 8, lines 138-140), but have not provided the statistics for each sex because this study did not focus on sex differences in any analyses.

Comment 2-4:

134-140. You here mention about special Japanese variant of corresponding scores. I think, you should provide concise description of differences them from international. In this context you should change abbreviations in the abstract.

Response:

Thank you for the comment. Following the comment, we have added relevant citations in the Methods section (page 11, lines 184-185). The MMSE-J and ADAS-J cog were officially translated to Japanese for the Alzheimer’s Disease Neuroimaging Initiative, which is an international collaboration project, and are widely used for both research and clinical medicine. The FAB-J has been validated as a translation of the original FAB and is widely used in Japan [4–6]. Therefore, we assume that there are no essential differences between the original and translated versions for all tests.

We apologise for the confusion caused by the description in the original abstract. We have modified it and explicitly described that we used their Japanese versions (page 2, lines 32-34).

Comment 2-5:

153 – abbreviation “meg” must be in uppercase.

Response:

Thank you for noting the typo. We have corrected it (page 15, line 255).

Comment 2-6:

In Methods you should describe hardware used for MEG abduction, protocol of this procedure, bands and channels you used for future analysis. The paper has complete lack of this technical information.

Response:

Thank you for the suggestion. Following the comment and comments by other reviewers, we have newly added subsections to describe the MEG data acquisition and analysis protocols (‘MEG data acquisition’ and ‘MEG analysis’ sections; pages 11-14, lines 191-239). We apologise for omitting these details in the original manuscript. Considering the topic of the present study, we assumed that few readers of this article would be interested in the details of the MEG techniques. However, the comments made us aware that these details are necessary for replications and required for scientific rigour. We hope that the necessary details are properly provided in these subsections; however, please kindly advise us again if you find that anything is still missing.

Comment 2-7:

I suppose a good idea would to provide short mathematical description of all parameters you analyze (MF, IAF, SSE).

Response:

Thank you for the comment. As replied to the previous comment (Comment #2-6), we have newly added a subsection to describe the MEG analysis protocols (‘MEG analysis’ section; pages 12-14, lines 201-239), where the explanations (including mathematical descriptions) of the MEG spectral parameters (i.e., MF, IAF, and SSE) have been also introduced.

Comment 2-8:

160-165. Provide mathematical formula for SOC value.

Response:

Thank you for the comment. We have added more detailed explanations regarding the SOC in the newly added subsection (‘Statistical analysis’ section; pages 15-16, lines 261-289). It is simply computed by dividing the group intercept (*β*_0i_) by the global slope (*β*_1_) of the linear regression model equivalent to the ANCOVA. We hope that the descriptions are sufficient to comprehend the SOCs, but we welcome further suggestions.

Comment 2-9:

173 – abbreviation “meg” must be in uppercase.

Response:

Thank you again for pointing this out. We have corrected it (page 18, lines 309-310).

Comment 2-10:

171 – 174. You write about significant differences in values. I think, you may provide here statistical results (p=…, etc.) mentioning the statistical method of evaluation.

Response:

Thank you for your suggestion. Following the comment, we have added the *p*-values in the main text (pages 17-18, lines 306-311). We intentionally omitted them from the original manuscript, because we listed them in Table 1. However, they were not properly displayed in the first version of the manuscript, due to a formatting error. We feel that the statistical values would provide support to the main text; thus, we have added them.

Comment 2-11:

181. Please provide more informative header of the table. What is P(unk)? In the text above (l. 173-179) you mention Spearman’s rank correlation but the table doesn’t provide any information about it. By the way the Table 1 provides information about groups of patients. I think you should include one more table with result of Spearman’s rank correlation analysis.

Response:

Thank you for the comments and suggestions. Following the comment and comments by other reviewers, we have modified the Results section and Table 1, and newly added S1 and S2 Tables (‘Group-level differences and correlations’ subsection; pages 17-18, lines 303-322). As correctly pointed out, Table 1 in the original manuscript was not informative because insufficient information was available to comprehend the contents (also, it seems that a few columns were missing at the right side of the table, possibly due to incorrect formatting). We apologise for this confusion. We have modified Table 1, so that it includes the results regarding the descriptive statistics of the parameters for each group (i.e., ‘Active’ and ‘Nonactive’) and their group-level comparisons using *t*-tests. Furthermore, we have added new tables (S1 and S2 Tables) to summarise the results of the correlation analyses. The results of the correlation analyses were described in the main text in the original manuscript; thus, they were omitted in the tables. However, it would be more comprehensible to summarise them in the table format; so we have added these tables in the revised manuscript. Please note that the statistical approach has been changed from nonparametric (Wilcoxon’s test and Spearman’s correlation) to parametric tests (*t*-test and Pearson’s correlations), given the large number of samples (N = 327) (pages 14-15, lines 241-260) and following a suggestion from another reviewer.

Comment 2-12:

Again, 188 - abbreviation “meg” must be in uppercase. Please look through the text and correct this error.

Response:

Thank you again. We apologies for the many typos. We have removed this line from the revised manuscript but have carefully proofread the whole manuscript to correct any remaining typos.

Comment 2-13:

188-191. Excuse me but I see no strong correlation between age and scores. There mostly are horizontal linear regression lines. I am strongly against “ritual” phrases in statistical reports (l. 191). If you mention ANOVA you should describe statistical model, you used, like one-way, two-way, repeated measurement and so on. Statistical results should have F and p values and information about post-hoc estimation of pair-wise comparison.

Response:

Thank you for pointing this out. Following the comments, we have modified the Results section (page 18, lines 312-314 and page 20, lines 332-334) and Table 2, and newly added S1 and S2 Tables. Regarding the correlation, we found no associations (i.e., correlational or regression relationships) between age and MEG spectral parameters. We did not address the correlational relationship between age and MEG spectral parameters in the original manuscript, where only limited pairs (within neuropsychological assessment scores and within MEG spectral parameters) had been subjected to the correlation analysis. We have modified the statistical strategy to address correlational relationships comprehensibly for all pairs (within and between neuropsychological assessment scores, MEG spectral parameters, and age) in the revised manuscript, which confirmed that age was not correlated with any MEG spectral parameters (i.e., MF, IAF, and SSE) but with the neuropsychological assessment scores (i.e., MMSE-J, FAB-J, and ADAS-J cog) for both groups (page 18, lines 312-314 and S1 and S2 Tables). This was replicated in the ANCOVA results, where the covariate (age) and its interaction to the group (i.e., ‘Active’ and ‘Nonactive’) were not significant for subjecting neither of the MEG spectral parameters (page 20, lines 332-334 and Table 2 (A)). These results correspond to the intuition that the least square lines in the scatterplot between age and MEG spectral parameters are close to horizontal (Fig 1).

We apologise for our misleading and insufficient descriptions regarding the statistical approach. We have modified the ‘Statistical analysis’ subsection (page 14-16, lines 241-289) thoroughly and described the full details of the ANCOVA and following SOC computations. The aim of the analysis was to assess the effect of group (‘Active’ vs. ‘Nonactive’) on the MEG spectral parameters while considering the effects of neuropsychological assessment scores. Therefore, we performed the ANCOVA with the MEG spectral parameters as dependent variables, group as independent variable, and covariance of neuropsychological assessment scores. Essentially, the ANCOVA is equivalent to the evaluation of the terms in a linear regression model using their variances and residuals; therefore, in our analysis environment (MATLAB), the ANCOVA and regression models are linked seamlessly by applying the ‘anova’ function on the estimated linear model. This caused our complicated descriptions in the original manuscript, in which we described that the linear regression models were ‘evaluated’ using ‘ANOVA’. Following this comment and comments by other reviewers, we have modified the explanations accordingly. The effect of group (‘Active’ vs. ‘Nonactive’) on the MEG spectral parameters was assessed by ‘ANCOVA’. The results are also summarised as a standard ANOCOVA table with *F*, *p*, and *η^2^* (effect size) in Table 2. There was only one two-level factor (group: ‘active’ and ‘nonactive’) with a covariance for each ANCOVA model; post-hoc tests / pairwise comparisons were not performed.

Comment 2-14:

202-209. From the text and table, I deduce that you use two-way ANOVA with two main factors 1) MEG value and 2) RPA to assess their influence on score parameter. I think, you should describe it in the text. Why do you not analyze the group of activity as impact factor?

Response:

Thank you for the question and comment. Following the comment, we have revised the ‘Statistical analysis’ subsection (pages 14-16, lines 241-289) and Results section thoroughly (pages 17-22, lines 302-362, Tables 1-2 and S1, S2 Tables). We apologise for the incomprehensible description regarding the ANOVA / ANCOVA models. As explained in the reply to the previous comment (Comment #2-13), our model included one of the MEG spectral parameters (i.e., MF, IAF, or SSE) as a dependent variable, group (i.e., ‘Active’ and ‘Nonactive’) as the independent variable, and one of the neuropsychological assessment scores (i.e., MMSE-J, FAB-J, and ADAS-J cog) as covariate. Although it was described as ‘ANOVA’ in the original manuscript, because of the expressions in the software (MATLAB) which we used for the statistical analysis, it is equivalent to ANCOVA in conventional statistical terms. We believe that the impacts of the group on the MEG spectral parameters have been assessed comprehensively using the *t*-tests and ANCOVA; however, we are open to further suggestions regarding more appropriate / superior strategies to examine the effects.

Comment 2-15:

210. Title of the table is bad. It describes statistical method. I think the title could give the reader idea what is estimated by the table. I would propose something from your text “Assessing the effects of regular physical activity on MEG spectral parameters”. And statistical method you could describe somewhere in Table note below.

Response:

Thank you for your constructive suggestion. Following the comment, we have gone through and modified the titles of the Tables and Figures. We hope that the new titles are appropriate in light of the journal guidelines; concise and descriptive.

Comment 2-16:

Better idea to introduce and describe Fig 2 in Results.

Response:

Thank you for the suggestion. We partially agree that Fig 2 does not sit well in the Discussion section. Therefore, we have modified and moved it to the Methods section, where the statistical model and SOC are introduced (pages 15-16, lines 261-289 and Fig 2), as it describes our idea of assessing the relationships between MEG spectral parameters, neuropsychological assessment scores, and groups. We hope that this modification improves the comprehensibility of the figure and that its embedding in the Method section is appropriate. Please confirm or let us know if you have better ideas.

Response to Reviewer 3 Comments

Comment 3-1:

Can the authors provide more details on the data acquisition (experimental paradigm and recordings= specifications) as well as the preprocessing (e.g., filtering) of the MEG data? The manuscript does not contain any information on this apart from descriptions on the three spectral outcomes.

Response:

Thank you very much for the suggestion. Following the comment and comments by other reviewers, we have newly added subsections to describe the MEG data acquisition and analysis protocols (‘MEG data acquisition’ and ‘MEG analysis’ subsections; pages 11-14, lines 191-239). We apologise for omitting these details in the original manuscript. Considering the topic of the present study, we assumed that few readers of this article would be interested in the details of the MEG techniques. However, the comments made us aware that these details are necessary for replications and required for scientific rigour. We hope that the necessary details are properly provided in these subsections; however, please kindly advise us again if you find that anything is still missing.

Comment 3-2:

The authors write that patients were classified as either ‘active’ or ‘nonactive’. The definition seems quite vague as it seems that patients reported these exercise levels themselves. Was this standardised in any way? For example, can the authors be more precise on what was meant with being physically active? Did the patients need to meet any criteria to be labelled active (e.g., in terms of frequency or duration)? Similarly, is it possible to have a more precise definition on what is meant with being 'nonactive'? If this is the most detailed information that the authors can provide, how sure are they that the ‘active’ and ‘nonactive’ groups are defined correctly?

Response:

Thank you for addressing this important point. Following to the comment, we have modified and added new information regarding the classification (‘Classification of Active and Nonactive groups’ subsection, pages 9-10, lines 158-179). We categorised patients into ‘Active’ and ‘Nonactive’ based on patients’ reports during clinical interviews on whether they have regular physical excises routines. We intentionally did not set thresholds for the classification across participants, such as types of activities (e.g., walking, swimming, playing golf, and trekking), duration (e.g., two hours per day), or frequency (e.g. every two days). There are two reasons for this. Firstly, there are no standard (i.e., widely accepted) criteria for being ‘active’. Sufficient amounts of physical activity should be defined in accordance with individual physical/mental health conditions. As the World Health Organisation mentioned on their homepage and guidelines, recommended levels of physical activity differ between individuals [7]. Light stretching could require huge efforts for some individuals with disabilities. It is not practical to engage in 150 minutes of vigorous-intensity aerobic physical activity per week for individuals with knee osteoarthritis. The appropriate level of physical activity should differ individually; thus, it cannot be categorised using common criteria. Secondly, this was an observational and retrospective study based on clinical records. The categorisation (i.e., ‘Active’ and ‘Nonactive’) was made post-hoc by reviewing the clinical records, but not at the time of the interview by asking standardised questions. Therefore, the descriptions regarding regular physical activity in the clinical records were highly heterogeneous; for example, some had a workout 30 min twice a day and others enjoyed bowling once a week.

Taken together, it is neither practical nor possible to quantify the amount of regular physical activity and evaluate (i.e., categorise) them objectively. It would be fair to categorise them with a binary criteria: whether they had a regular exercise routine or not. If the patients reported during their medical interviews that they had a regular exercise routine, they were assigned to the ‘Active’ group; otherwise, they were assigned to the ‘Nonactive’ group. Of note, if there was no record regarding regular physical activity, the patients were excluded from the study.

Comment 3-3:

Line 154 and 173, and 188: meg -> MEG

Response:

Thank you for noting the typo. We have thoroughly proofread and revised the manuscript to ensure that remaining typos were corrected.

Comment 3-4:

Line 171 and 181: How is it possible that the p-values are different between the in-text report (line 171; p = 0.647) and Table 1 (line 181; p = 0.305)?

Response:

Thank you for pointing this out. Following the comment, we have modified the Results section (pages 17-18, lines 306-311) and Table 1. We apologise for the confusion. The mismatch was possibly due to minor changes in the analysis pipelines during manuscript writing and/or incorrect formatting (cutoff of a few columns) in Table 1 in the original manuscript. In the revised manuscript, following the comment from another reviewer, we have changed the statistical analysis; the statistical approach has been changed from nonparametric (Wilcoxon’s test and Spearman’s correlation) to parametric tests (*t*-test and Pearson’s correlations) given the large number of samples (N = 327) (page 14, lines 243-244). The results (with identical *p*-values) are reported in the main text (pages 17-18, lines 306-311) and Table 1.

Comment 3-5:

Line 181 (Table 1): Can the authors please report test statistics, degrees of freedom and effect sizes for these statistical contrasts?

Response:

Thank you for your suggestions. Following the comment, we have modified the Tables (Tables 1 and 2, and S1 and S2 Tables), including the test statistics (*T*, *r*, or *F*), degree of freedom (only for the two-sample *t*-test in Table 1), and effect sizes (Cowen’s *d*, *r*, or *η*^2^) along with the *p*-values. We apologies for omitting these details. In Table 1 in the original manuscript, a few columns seem to have been cutoff at the right part of the Table, where the test statistics were reported.

Response to Reviewer 4 Comments

Comment 4-1:

The purpose of the first paragraph is unclear. Is it intended to define dementia, explain why specific tests were used, or discuss the difficulty of predicting cognitive decline in dementia patients? Clarify the focus.

Response:

Thank you for your constrictive comment. Following the comment, we have revised the Introduction section. The goal of the present study is to clarify whether neurophysiological parameters (i.e., MEG spectral parameters) are influenced by healthy lifestyles, while controlling for current cognitive states (i.e., neuropsychological assessment scores), which is a clinically important question. Leading to the introduction of MEG and lifestyle factors, we organised the first paragraph to generally introduce the targeting disease, dementia, and its primary characteristics, a symptomatic nature, which are usually evaluated by neuropsychological assessments. Neither MEG nor physical exercises are mentioned in the first paragraph, which might still give the impression that the section lacks focus. However, as we have thoroughly reorganised the Introduction section, we would like the reviewer to re-evaluate it and remain open to further suggestions (if any).

Comment 4-2:

The opening sentence of the second paragraph makes a broad claim but lacks references to support it. Provide appropriate citations.

Response:

Thank you for your insightful comment. We assume that the comment was targeting the sentence ‘Regular physical activity is a prominent factor that contributes to a better prognosis of cognitive decline’, which was presented without references in the original manuscript. Although we intended to support the sentence by the following sentences and presented it as a summary of the paragraph, we agree with the reviewer that the claim might be too broad. After a thorough revision, the corresponding part was eliminated from the manuscript.

Comment 4-3:

The concept of "functional reserve" should be explained before introducing it.

Response:

Thank you for pointing this out. To make the manuscript more focused and clearer, we have removed the concept of ‘functional reserve’ from the revised manuscript. A similar concept, ‘cognitive reserve’ is introduced in the Discussion section (pages 25-26, lines 416-431) accompanied by a detailed definition.

Comment 4-4:

This paragraph appears to discuss the role of physical activity, but the connection to cognitive reserve is unclear. Use examples that directly relate to cognitive reserve (e.g., mental stimulation, education) rather than unrelated analogies like kidney/liver functions.

Response:

Thank you for this important comment. Following this and other comments, we have thoroughly revised the Introduction section. The concept of ‘cognitive reserve’ is now introduced in the Discussion section (pages 25-26, lines 416-431), where its physiological relevance is described extensively. We hope that the revised manuscript provides clear links between neurological changes and cognitive reserve.

Comment 4-5:

Explain the physiological mechanisms by which physical activity might prevent cognitive decline, with appropriate references to support these claims.

Response:

Thank you for your suggestion. Following the comment, we have added descriptions regarding the hypothetical mechanisms how physical activity contributes to cognition (pages 23, lines 375-383). It is considered that there are two different mechanisms underlying the link: direct and indirect pathways. Regarding the direct pathway, physical activity improves blood flow, reduces inflammation, induces synthesis and releases neurotrophins, which modulate amyloid β turnover and consequently improve cognition [8]. As the indirect pathway, regular exercise routines reduce other risk factors, such as hypertension [9], obesity [10], and diabetes [11], which contributes to preventing cognitive decline. Following the reorganisation of the Introduction section, we have added these detailed descriptions in the Discussion section (pages 23, lines 374-383). We hope that these explanations help the readers to comprehend the background of the present study and our interpretations of the results.

Comment 4-6:

In the third paragraph the claim that electrophysiological measurements do not correlate well with cognitive assessment may reflect issues with the measurements themselves, not necessarily the impact of cognitive reserve due to physical activity. Avoid oversimplified assumptions and provide a robust explanation.

Response:

Thank you for your constructive comment. We agree and admit that our argument did not sound very reasonable. Following the comment, we have reconsidered the relationships between electrophysiological measurements and physical activities, described them in the Introduction section (pages 6-7, lines 96-113), and newly added Fig 1. Previous studies have shown that MEG spectral parameters are well associated with both cognitive state [2] and pathological state [12–16]. Regular physical activity influences broad parts of the neurological state including physiological and pathological states [17–21]. Thus, we hypothesised that the MEG spectral parameters would be indicators of the neurological changes triggered by lifestyle-associated factors, and that they would differ between patients with different lifestyles as well as correlate with neuropsychological assessment scores. We hope that the revised manuscript expresses our concept clearly.

Comment 4-7:

Explain how spectral measurements are proposed to demonstrate cognitive reserve. Clarify why cognitive reserve might not directly improve cognitive performance but could indirectly affect spectral measures.

Response:

Thank you for this insightful comment. Accordingly, we have added descriptions regarding the possible mechanisms underlying the links between physical activity, cognitive reserve, and the MEG spectral parameters (page 25-26, lines 416-431). Importantly, cognitive performance is a gross output of patients’ neurological conditions, including their pathological and physiological states, which is modulated by lifestyle-associated factors (Fig 1). Therefore, we suppose that the MEG measurements would capture the neurological changes related to physical activity.

Comment 4-8:

The hypothesis that electrophysiological activity represents both functional reserve (via physical activity) and cognitive state independently is unclear. Provide a logical connection between these concepts and how they are tested in this study.

Response:

Thank you for your constructive comment. Following this and previous comments (Comments 4-1 to 4-7), we have revised the Introduction and Discussion sections thoroughly and added new Fig 1. We have introduced the links between lifestyle-associated factors (e.g., physical activity), neurological states (e.g., MEG measurements), and cognitive states (e.g., neuropsychological measurements) clearly and logically in the Introduction section (pages 6-7, lines 96-113). The concept of ‘functional reserve’ has been removed from the revised manuscript to simplify the storyline, while that of ‘cognitive reserve’ was only discussed in the Discussion section, along with a clear definition (pages 25-26, lines 416-431). We believe that these changes improved the logics behind our study; however, we are open to further suggestions if the reviewer finds it unsatisfying.

Comment 4-9:

Overall, the introduction would benefit from a more coherent structure, improved clarity, and a less exaggerated presentation of the hypothesis.

Response:

Thank you very much for your constructive and educative comment. Following this and previous comments, we have re-organised and thoroughly revised the Introduction section. To enhance its comprehensibility, we have also added Fig 1. We hope that these changes yielded a more suitable and clearer introduction.

Comment 4-10:

Dataset: Define the threshold used to classify participants as cognitively healthy.

Response:

Thank you for the comment. We believe that ‘cognitively healthy’ should not be defined by fixed thresholds, practically and legally. It is a matter of diagnosis, which is based on comprehensive assessments and interviews, but does not simply depend on a single or few objective measurement(s) [22]. Here, we used the phrase ‘cognitively healthy’ to explain the procedure for obtaining informed consent (page 9, lines 151-153). This was a retrospective observational study using a large clinical dataset; thus, the participants were not research volunteers but clinical patients. We, an attending physician, a support doctor, clinical psychologists, and clinical laboratory technicians, evaluated the patients based on their clinical experiences and judged whether they were competent to consent for participation. In practice, we always ask both the patients and their legal guardians for written consent to participate in clinical studies, except for some special circumstances. To clarify this, we have added a description in the Methods section (page 9, lines 151-153).

Comment 4-11:

Parameters: Avoid claiming that "these parameters are established in clinical practice" if this assertion is based on a single study, and a case serios, particularly if conducted by the same group. Broader validation is necessary to support this statement.

Response:

Thank you for your comment. We agree that they were only validated in a limited environment. Following the comment, we have modified our expressions (page 12, lines 202-204, and pages 13-14 lines 236-239). To the best of our knowledge, only three hospitals are using these parameters for the clinical practice.

Comment 4-12:

Parameters: Define "regular exercise routine" explicitly. Was this based on general responses (e.g., "Do you exercise regularly?") or specific criteria (e.g., "Do you walk 30 minutes at least three times per week?"). If the former, this grouping may lack reliability.

Response:

Thank you for your important comment. Following this and comments by other reviewers, we have added a new subsection to describe the classifications in detail (‘Classification of Active and Nonactive groups’ subsection; pages 9-10, lines 158-179). As described in a guideline published by the World Health Organisation [23], appropriate amounts of or types of physical activity highly depend on patients’ backgrounds. It is not appropriate nor practical to classify the patients as ‘Active’ or ‘Nonactive’ using a specific quantifiable threshold (e.g., 30 min walk at three times per week) without considering their backgrounds (e.g., patients are in a wheelchair or suffer from dizziness). For example, even light body stretching requires huge efforts for some patients and we often observe that such activities improve their cognition. Thus, we intentionally did not set detailed rules and./or quantifiable thresholds for the classification. This is consistent with the concept of person-centred care [24], which is an essential attitude in clinical medicine.

Comment 4-13:

Statistics: Justify the use of Wilcoxon tests. For a dataset of this size, a t-test or ANOVA would be more appropriate and straightforward.

Response:

Thank you for your suggestion. Indeed, for this sample size (N = 327), parametric test would be the straightforward option. Accordingly, we have modified the statistical approach from nonparametric (i.e., Wilcoxon test and Spearman’s correlation) to parametric (i.e., *t*-test and Pearson’s correlation) tests throughout the analysis, and modified the ‘Statistical analysis’ subsection (page 14, lines 241-254) and Results section (pages 17-18, lines 306-322).

Comment 4-14:

MEG analysis: There were no mention of how the analysis of MEG data have been done, the preprocessing of the data as well as the calculation of spectral parameters should be explained in details.

Response:

Thank you very much for pointing this out. Following this and comments by other reviewers, we have added new subsections to describe the details of the MEG data acquisition and analysis (‘MEG data acquisition’ and ‘MEG analysis’ subsections; pages 11-14, lines 191-239). We apologise for omitting these details in the original manuscript. Considering the topic of the present study, we assumed that few readers of this article would be interested in the details of the MEG techniques. However, the comments made us aware that these details are necessary for replications and required for scientific rigour. We hope that the necessary details are properly provided in these subsections; however, please kindly advise us again if you find that anything is still missing.

Comment 4-15:

Correlations: The manuscript reports significant correlations between cognitive scores and cognitive measures, as well as between MEG measures and MEG measures, but fails to explore correlations across these domains (e.g., MEG measures with cognitive scores). This should be addressed.

Response:

Thank you for the comment. Following this, we have performed the correlation analyses comprehensively within/between age, MEG spectral parameters, and neuropsychological assessment scores (page 14, lines 248-252). We apologise for the mismatch between the Methods (in the ‘Statistical analysis’ subsection, we described that ‘intercorrelations between the neuropsychological scores and MEG spectral parameters’ were examined) and Results sections (only intra-correlations within neuropsychological scores and within MEG spectral parameters were reported) in the original manuscript. We have thoroughly revised the manuscript and reported the results of all correlation analyses in the Results section (page 18, lines 311-322) and S1 and S2 Tables.

Comment 4-16:

Evaluation of Regression Models and Physical Activity Effect: These two sections are unclear to me. Clarify the methods and results to make the findings interpretable.

Response:

Thank you for the comment. Following this and comments by other reviewers, we have thoroughly modified the ‘Statistical analysis’ subsection (pages 14-16, lines 241-289) and Results section (pages 17-22, 302-362), Fig 2, and Table 2.

Firstly, we apologise for the insufficient information that was provided with poor comprehensibility in the original manuscript. We have used the regression strategy to assess the effect of group (‘Active’ vs. ‘Nonactive’) on the MEG spectral parameters while considering the effects of neuropsychological assessment scores. Therefore, we performed the ANCOVA with the MEG spectral parameters as dependent variables, group as independent variable, and covariance of neuropsychological assessment scores. Essentially, the ANCOVA is equivalent to the evaluation of the terms in a linear regression model using their variances and residuals; therefore, in our analysis environment (MATLAB), the ANCOVA and regression models are linked seamlessly by applying the ‘anova’ function on the estimated linear model. This caused our complicated descriptions in the original manuscript, in which we described that the linear regression models were ‘evaluated’ using ‘ANOVA’. Following this comment and comments by other reviewers, we have modified the explanations accordingly. The effect of group (‘Active’ vs. ‘Nonactive’) on the MEG spectral parameters was assessed by ‘ANCOVA’. The results are also summarised as a standard ANOCOVA table with *F*, *p*, and *η^2^* (effect size) in Table 2.

The regression model was used for computing the SOC. The SOC is the quantification of the impact of regular physical activity (i.e., group) on the MEG spectral parameters in a unit of neuropsychological assessment scores. To make the group and neuropsychological assessment scores comparable, we have estimated the coefficients of the linear regression model, which was equivalent to the ANCOVA model. The SOC was computed as a relative size (ratio) of the group intercept against the slope of the covariate. For this purpose, we have described the regression model with a formula and other sufficient information (page 15-16, lines 261-289). Furthermore, we have moved Fig 2 to the Methods section to describe the statistical model and SOC supplementarily, schematically, and intuitively. We hope that these explanations of the statistical methods and results are clear in the revised manuscript.

Comment 4-17:

The statement that "[MEG spectral parameters] indicate a functional reserve that may help prevent future cognitive decline, regardless of the current cognitive state" is not supported by the findings. How was cognitive reserve measured in this study?

Response:

Thank you for your comment. Following this and previous comments (Comment 4-1, 4-2, 4-3, and 4-4), we have thoroughly changed the Introduction and Discussion sections. Consequently, the concept of ‘functional reserve’ was eliminated (the sentence in the comment was also deleted) to simplify the storyline. Based on the obtained results, we have introduced the ‘cognitive reserve’ in the Discussion section (pages 25-26, lines 416-431), which would be one of the interpretations of the neurological fingerprint of physical exercise. We hope that these interpretations are reasonable for the readers.

Comment 4-18:

The conclusions drawn from the results are overgeneralized and not sufficiently supported. Avoid overinterpreting simple correlations.

Response:

Thank you for the important comment. We agree that the conclusion should be made appropriately while avoiding oversimplifications. Following the comment and all changes which we made in the revised manuscript, we have modified the conclusion (page 28, lines 460-464).

Comment 4-19:

The discussion is overly focused on functional reserve, a concept that appears unrelated and untested within the study. This weakens the narrative and risks misrepresentation of the findings.

Response:

Thank you for this insight which made us aware that the concept of a functional reserve does not explain the links between physical exercise and neurological states very well; we have removed the concept and refined the logics behind the relationships, which are mainly described in the Introduction section and Fig 1. We would appreciate further comments regarding these revisions.

Comment 4-20:

To demonstrate that physical activity prevents cognitive decline, the study should begin by establishing a correlation between the amount of physical activity and cognitive scores or show a significant difference in cognitive scores between active and inactive groups. The relevance of MEG measurements is unclear in this context.

Response:

Thank you for the constructive comment. We apologise that our motivations were introduced well in the original manuscript. We have thoroughly rewritten the Introduction and Methods sections to describe the purpose and our strategy to examine the hypothesis clearly. The goal of the present study was to clarify whether neurological parameters (i.e., MEG spectral parameters) are influenced by a healthy lifestyle, while controlling for current cognitive states (i.e., neuropsychological assessment scores), which is a clinically important question. We used *t*-tests and ANCOVA to show that MEG spectral parameters were different between patients with and without regular physical activity (i.e., ‘Active’ and ‘Nonactive’ groups), which supported our hypothesis. We hope that the revised manuscript conveys our message clearly.

Comment 4-21:

To support the claim that physical activity prevents cognitive decline, the manuscript should first establish a direct correlation between physical activity and cognitive scores. Alternatively, compare cognitive scores between physically active and inactive groups.

Response:

Thank you again for your comment. As replied to the previous comment (Comment #4-20), the effect of physical activity on cognitive decline itself is not a main topic of the present study. The goal of the present study was to clarify whether neurological parameters (i.e., MEG spectral parameters) are influenced by a healthy lifestyle, while controlling for current cognitive states (i.e., neuropsychological assessment scores), which is a clinically important question. Nonetheless, we have demonstrated the differences in neuropsychological assessment scores between the groups using *t*-tests (pages 17-18, lines 306-311 and Table 1). We hope the point is clear in the revised manuscript.

Comment 4-22:

The inclusion of MEG measurements seems disconnected from the primary research question. If MEG is to be used, consider using logistic regression to predict activity status (active vs. inactive) based on MEG and cognitive measures. Then, test whether including MEG measurements improves predictive power.

Response:

Thank you for the constructive suggestion. If the primary research question was to examine the effects of physical activities on cognitive performance, we could assess the MEG data supplementarily and examine their contributions to the predictive performance, as the reviewer suggested. However, as we replied to the previous comments (Comment #4-20 and #4-21), the purpose of the study was to examine the influence of regular physical activity on neurological states (i.e., MEG spectral parameters), which is a clinically important question. Therefore, although we agree that the logistic regression and predictions of the groups using MEG parameters are interesting topics that should be addressed, they are beyond the scope of the present study.

Comment 4-23:

Simplify statistical analyses. The use of overly complex models for simple comparisons detracts from clarity and accessibility.

Response:

Thank you for the important comment. We assume that the complexity that the reviewer found in the original manuscript was due to our poor description of the statistical analysis procedure, particularly regarding the later parts (i.e., ‘regression analysis’ followed by ‘ANOVA’ and ‘SOC’ in the original manuscript). As explained in the reply to comment #4-16, the purpose of our approach was to examine the effect of group (‘active’ vs. ‘nonactive’) on the MEG spectral parameters while considering the effects of neuropsychological assessment scores. For this purpose, only ANCOVA would be sufficient. The strategy used in the original manuscript (i.e., ‘regression analysis’ and following ‘ANOVA’) was equivalent to ANCOVA, but the description was complicated as it followed the expressions used in our analysis software (MATLAB). Therefore, in the revised manuscript, we have thoroughly modified the ‘Statistical analysis’ subsection (pages 14-15, lines 241-260) using ‘ANCOVA’. The regression analysis that we used for computing SOCs has been explained much more extensively (pages 15-16, lines 261-289) and supplemented with schematic figure (Fig 2). We hope that the revised analysis methods are deemed sound and comprehensible.

Comment 4-24:

The manuscript has potential but requires substantial revisions to address the issues outlined above. The introduction needs a more focused narrative, the methods and statistical analyses need better justification, and the results should be clarified and aligned with the hypotheses. Furthermore, the conclusions should be restrained to reflect the actual findings without overstating their implications.

Response:

Thank you very much for your positive evaluation and detailed comments. Your comments helped us to significantly improve the quality of the manuscript. Consequently, we have revised almost the whole manuscript. We hope that you deem the new manuscript improved and acceptable for the publication.

Response to Reviewer 5 Comments

Comment 5-1:

Major issue: The manuscript lacks any kind of detail about how MEG data was collected, preprocessed and analyzed. Without such information, it is impossible to establish whether MEG data has been treated according to scientific conventions and whether the MEG analysis pipeline could have influenced the current results. Because MEG spectral measures are a central part of the current study, the authors should have included a detailed subsection to within their methods section explaining each step in the MEG data analysis pipeline. Such section should include, among other things: the MEG system used, the manufacturer, whether online filters were applied, what instructions were given to participants, whether data was acquired in eyes-closed or eyes-open resting-state conditions, what package was used to analyze MEG data, off-line filters, procedures to identify bad channels or noisy segments, whether these were manual or automatized, whether data was epoched or not, and if so, what was the epoch’s duration, whether epochs were rejected, and if so, what epoch rejection thresholds or parameters were used, the total number of epochs before and after epoch rejections per individual, among others.

Similarly, regarding MEG data analysis, the authors need to provide a detailed description of how spectral parameters were obtained, describing all relevant steps, methods, tools used in their signal-processing pipeline and mathematical formulas (if necessary). For instance, it is not clear what type of method was used to compute the spectral representation of the time-domain signal, what parameters were set for such analysis, and how the spectral measures were subsequently estimated. An example of how to report both MEG preprocessing and spectral analyses can be found in references 20 and 23 of the manuscript, or in other similar papers by that same group.

Response:

Thank you for your detailed comment. Following this and comments by other reviewers, we have added new subsections to describe the details of the MEG data acquisition and analysis (‘MEG data acquisition’ and ‘MEG analysis’ subsections; pages 11-14, lines 191-239). We apologise for omitting these details in the original manuscript. Considering the topic of the present study, we assumed that few readers of this article would be interested in the details of the MEG techniques. However, the comments made us aware that these details are necessary for replications and required for scientific rigour. We hope that the necessary details are properly provided in these subsections; however, please kindly advise us again if you find that anything is still missing.

Comment 5-2:

Major issue: There are serious issues with how statistical analyses are reported. For instance, the authors report their correlation analyses and direct the reader to table 1 [line 179], but I believe table 1 actually presents the results of the Wilcoxon Signed Rank Tests. Relatedly, the authors state that Fig 1 shows the regression analyses [line 188], but the title of Fig1 indicates these scatter plots represent the correlation between spectral parameters and neuropsychological assessment. It is therefore unclear what analysis figure 1 actually shows.

The regression analyses must be described and reported in full detail, but instead, the authors provide almost no information about the results of their regression analyses in their result section, other than referring the reader to figure 1. First of all, authors need to explicitly state how models were specified (provide their formulas). For instance, the author’s state:

“We then assessed the effect of regular physical activity on meg spectral parameters while accounting for cognitive state. Each meg spectral parameter (MF, IAF, and SSE) was subjected to a multiple regression model with three predictors: (1) a categorical predictor of regular physical activities (‘Active’ and ‘Nonactive’), (2) age or one of the neuropsychological assessment scores (MMSE-J, FAB-J, or ADAS-J cog) as a covariate, and (3) their interaction term.” [Lines 152-157].

Based on this, I would assume that the models took the form:

y ~ RPA + AGE + RPA*AGE

y ~ RPA + MMSE-J + RPA*MMSE-J

y ~ RPA + FAB-J + RPA*FAB-J

y ~ RPA + ADAS-J + RPA*ADAS-J

This is just an assumption, and I do not know if this is correct, which illustrates the problem: there is very little information about how many models were designed and how they were specified. Without this information, it is very difficult to assess whether models were appropriate and whether the author’s interpretation of their results are justified.

Regression tables could be used to summarize the results, illustrating whether the effect of each term within each model is actually significant or not. Alternatively, this information could be presented within the text. Regardless, it is crucial that the reader understands whether factors or their interaction have a significant effect in predicting changes in the dependent variable. Such information, however, is not provided.

Response:

We appreciate the detailed review and suggestions. Following the comment, we have thoroughly modified the ‘Statistical analysis’ subsection (pages 14-16, lines 241-289) and Results section (pages 17-21, lines 302-356). Firstly, we apologise for the incorrect references to Tables and Figures. As pointed out, Table 1 shows the descriptive statistics and the results of group-level comparisons (‘Active’ vs. ‘Nonactive’). The results of the correlation analysis were not described in the table but only appeared in the main text for limited pairs of parameters (the correlations ‘within’ MEG spectral parameters and ‘within’ neuropsychological assessments, but not ‘between’ them) in the original manuscript. We have added descriptions regarding the correlation analysis in the ‘Statistical analysis’ subsection (pages 14-15, lines 241-260) and reported the results in the newly added tables (S1 and S2 Tables). Also note that the statistical approach has been changed from nonparametric (Wilcoxon’s test and Spearman’s correlation) to parametric tests (*t*-test and Pearson’s correlations) given the large number of samples (N = 327) (page 14, lines 243-244) and following a suggestion from another reviewer.

Regarding the regression analysis, we apologise for the confusion. We have used the regression strategy to assess the effect of group (‘active’ vs. ‘nonactive’) on the MEG spectral parameters while considering the effects of neuropsychological assessment scores, but not for assessing the validity of the models or for prediction performance. Therefore, we performed the ANCOVA with the MEG spectral parameters as dependent variables, group as independent variable, and covariance of neuropsychological assessment scores. Essentially, the ANCOVA is equivalent to the evaluation of the terms in a linear regression model using their variances and residuals; therefore, in our analysis environment (MATLAB), the ANCOVA and regression models are linked seamlessly by applying the ‘anova’ function on the estimated linear model. This caused our complicated descriptions in the original manuscript, in which we described that the linear regression models were ‘evaluated’ using ‘ANOVA’. Following this comment and comments by other reviewers, we have modified the explanations accordingly. The effect of group (‘Active’ vs. ‘Nonactive’) on the MEG spectral parameters was assessed by ‘ANCOVA’. The results are also summarised as a standard ANOCOVA table with *F*, *p*, and *η^2^* (effect size) in Table 2.

The regression model was used for computing the SOC. The SOC is the quantification of the impact of regular physical activity (i.e., group) on the MEG spectral parameters in a unit of neuropsychological assessment scores. To make the group and neuropsychological assessment scores comparable, we have estimated the coefficients of the linear regression model, which was equivalent to the ANCOVA model. The SOC was computed as a relative size (ratio) of the group intercept against the slope of the covariate. For this purpose, we have described the regression model with a formula and other sufficient information (pages 15-16, lines 261-289). Furthermore, we have moved Fig 2 to the Methods section to describe the statistical model and SOC supplementarily, schematically, and intuitively. We hope that these explanations of the statistical methods and results are clear in the revised manuscript.

Comment 5-3:

Major issue: To properly evaluate model performance, authors should also report metrics of goodness-of-fit for each model, such as R2, AIC, or BIC for each model. Unfortunately, none of these values are reported. The authors report SOC (Size of Contribution) as a measure of how much one factor contributes to model performance, but this is not enough to back up the interpretations they derive from their regression analyses in their discussion section. I am also having difficulties understanding table 2. The authors state that

“To evaluate the regression models assessing the effects of regular physical activity on MEG spectral parameters while accounting for age or one of the neuropsychological assessment scores, we performed an ANOVA (Table 2)”.

Based on this, I would expect a stepwise model comparison procedure where covariates are sequentially added to initial models and then the statistical effect of adding such covariates is assessed via maximum likelihood estimation. Instead, the authors present table 2 as a summary of their model comparison procedure. However, it is difficult to understand from table 2 how models were compared and how the effect of covariates was assessed. Actually, table 2 gives the impression that spectral parameters were treated as predicting (i.e. independent) variables because they are reported with associated F and p values, as if they were factors within a new set of independent models. It is therefore not clear how different models were compared, or how the presence or absence of their covariates of interest affected their models.

Response:

We thank the reviewer for raising these concerns. First, as clarified in the reply to the previous comment (comment #5-2), we did not aim to compare the models or to evaluate the prediction performances. We simply wanted to assess the impact of group (i.e., ‘Active’ vs. ‘Nonactive’) on the MEG spectral parameters while considering the neuropsychological assessment scores as covariates. Based on the pipeline of the statistical analysis for this purpose, we described the linear regression model followed by the evaluation of the terms (following the pipeline in the MATLAB software where the ‘anova’ function was applied to the regression model), which is equivalent to ANCOVA. We have modified the explanation using ‘ANCOVA’ (pages 14-15, lines 254-260) instead of introducing the regression models, which should be more comprehensible and straightforward for readers.

Regarding the SOC, we apologise for the insufficient explanation in the original manuscript. Following this comment and comments by other reviewers, we have revised the descriptions (pages 15-16, lines 261-289). We computed it to quantify the impact of regular physical activity (i.e., group) on the MEG spectral parameters in a unit of neuropsychological assessment scores. Therefore, it is unrelated to the model performance. It was calculated using the estimated coefficients of the linear regression model, which is equivalent to the ANCOVA model where the main effect of the group (i.e., independent variable, or categorical predictor in the modelling term) was significant. The regression model was equivalent to the ANCOVA model, and we did not aim to refine or evaluate it to improve performance. Therefore, model performance indicators, such as R2, BIC, and AIC, were not considered, and a stepwise comparison was not performed either.

We hope that the modifications in the Statistical analysis and Results sections are reasonable and provide sufficient information. However, please do not hesitate to raise remaining issues regarding the analysis strategies and reporting styles.

Comment 5-4:

Major issue: A somehow worrisome issue at the conceptual level is the use of ambiguous terminology in the introduction. Throughout this section, the authors use two concepts, functional reserve and cognitive reserve, which are not mutually interchangeable. Functional reserve is physiologically quantifiable, while cognitive reserve is behaviorally, rather than physiologically quantifiable. I understand the authors attempt to establish a parallelism between functional and cognitive reserve. However, in later sections of the manuscript, it remains unclear whether the authors are empirically concerned with functional reserve (which might be quantifiable via MEG), cognitive reserve (which would be quantifiable via neuropsychological assessment), or both. What is more, the authors later state:

“We hypothesized that electrophysiological brain activity would represent both the amount of functional reserve (i.e., regular physical activity) and the current cognitive state independently” .

This implies that regular physical activity is their operationalization of functional reserve. Such a lack of conceptual clarity makes it difficult to connect relevant concepts with their operationalized independent variables of interest. Along with the aforementioned issues in how regression models are specified and reported, it is very difficult to assess the validity of the interpretations offered by authors in the discussion section of their work.

Response:

Thank you for pointing out the issues regarding the concepts in the original manuscript. We understand the concerns and reorganised the whole manuscript accordingly; the major changes in the conceptual levels are as follows:

1. We have removed the concept of ‘functional reserve’ from the manuscript to simplify the storyline.

2. We have positioned the concept of ‘cognitive reserve’ as one of the potential interpretations of the results—the neurological fingerprint of the physical activity—and introduced it in the Discussion section (pages 25-26, lines 416-431).

3. In the Introduction section, we have refined the logics behind the purpose of the present study; to examine the influence of regular physical activity (i.e., one of the lifestyle-associated factors) on neurological states (i.e., MEG spectral parameters) while controlling for effects of cognitive states (i.e., neuropsychological assessment scores) (pages 6-7, lines 96- 113).

We believe that the revised manuscript includes valid conceptual ideas and would appreciate your opinions on it.

Comment 5-5:

Other issue: It would be useful if the authors offered a more detailed description of “cognitive states”, especially considering the broad scope of the journal. I assume that when they use the term “cognitive state”, they refer to the clinical assessment of multiple cognitive functions such as attention, memory, alertness, among others, as quantified by clinically-validated neuropsychological questionnaires and assessment tools.

Response:

Thank you for the constructive suggestion. Accordingly, we have added clear descriptions regarding the ‘cognitive states’ to the revised Introduction section (page 3-4, lines 53-57). We hope that this helps readers to comprehend the background of the present study.

Comment 5-6:

Other issue: The authors are right that MEG measures the magnetic fields produced by the electrophysiological activity of populations of neurons in the cerebral cortex, but it is inaccurate to state that MEG reflects dementia-associated cognitive states [Lines 79-81]. MEG cannot directly reflect cognitive states, but rather, provide measures of brain function that might (or might not) be indicative of those states. Relatedly, while the authors do well in justifying their three spectral measures of interest, they should explicitly acknowledge that these here are just a few among many more parameters and measures of brain function that can be obtained using MEG.

Response:

Thank you for your constructive comments. Following the comments, we have modified the introductions of the MEG techniques (pages 5-6, lines 80-91). First, we agree with the reviewer that MEG parameters are not direct reflections of cognitive states, as they measure the brain activities which are related to cognition. In the revised manuscript, we tried to be as accurate as possible regarding this relationship. Next, regarding the choice of parameters (i.e., MF, IAF, and SSE), the reviewer correctly pointed out that MEG data can be summarised in various ways which may be indicators of cognitive states. We explicitly acknowledge that these three, out of many possible others, were the focus of the present study.

Comment 5-7:

Other issue: The authors state that “This study revealed that regular physical activity significantly influenced electrophysiological brain activity while controlling for the current cognitive state (i.e., neuropsychological scores). This influence was significant across any neuropsychological assessment scores considered”, but considering the lack of clarity on MEG and statistical analysis, it is hard to see how they arrived at this conclusion. This might also be an overinterpretation of their results, particularly because MEG-measured brain dynamics during resting state are known to be influenced by a myriad of genetic and environmental factors, which are not controlled within the current experimental design.

Response:

Thank you for this comment. We interpreted that the comment pointed out two issues. First, the descriptions regarding MEG and statistical analyses were insufficient in the original manuscript. As replied to the previous comments (Comment #5-1, #5-2, and #5-3), we have added detailed descriptions of the MEG acquisition and analysis and refined the explanations of the statistical procedures (pages 11-16, lines 191-289). We believe that these modifications provide sufficient information to evaluate the results properly.

Second, the reviewer is concerned about the presence of confounding factors, such as genetic, environmental, and other lifestyle-associated factors. Following the comment, we have clarified the potential contributions of these factors in the present study (pages 27-28, lines 450-458). We have mentioned that many lifestyle-associated factors have been identified, such as less education, head injury, physical inactivity, smoking, excessive alcohol consumption, hypertension, obesity, diabetes, hearing loss, depression, infrequent social contact, and air pollution [17]. We focused on a regular exercise routine as a representative index of lifestyle-associated factors, because it is modifiable and clinically important. Importantly, while considering the regular exercise routine as a main interest, we did not rule out the influences of other factors, because they are not separable. All lifestyle-associated factors interact with each other and contribute synergically to reduce (or increase) the risk of cognitive impairment. For example, a regular exercise routine contributes to reducing the risk of cognitive decline via reducing the risk of hypertension and/or depression. Therefore, we considered a regular exercise routine as a gross indicator of all lifestyle-associated factors in the present study. The reviewer also mentioned other confounding factors, which are not lifestyle associated: genetic and environment factors. These factors could influence MEG spectral parameters independently from regular physical activity. In such cases, we cannot address them because this was a retrospective study based on existing clinical records, and details regarding these factors were not available. We have added this as the third limitation in the revised manuscript (pages 27-28, lines 450-458).

Even under these constrains, the present results are clinically valuable because we are interested in prospectively modifiable factors. Regular physical activity is a typical one and its influence can be evaluated using MEG spectral parameters.

Comment 5-8:

Other issue: In the same vein, an important covariate that the authors did not include in their analyses was educational attainment. This is important because the authors cite previous research linking educational attainment with cognitive reserve (Stern et al. 1992), and previous studies investigating the effect of physical activity on MEG-measured brain activity have in fact included educational attainment as a control variable (de Frutos-Lucas et al. 2018).

de Frutos-Lucas, J., López-Sanz, D., Zuluaga, P., Rodríguez-Rojo, I. C., Luna, R., López, M. E., Delgado-Losada, M. L., Marcos, A., Barabash, A., López-Higes, R., Maestú, F., & Fernández, A. (2018). Physical activity effects on the individual alpha peak frequency of older adults with and without genetic risk factors for Alzheimer’s Disease: A MEG study. Clinical Neurophysiology, 129(9), 1981–1989. https://doi.org/10.1016/j.clinph.2018.06.026

Stern Y, Alexander GE, Prohovnik I, Mayeux R. Inverse relationship between education and 402 parietotemporal perfusion deficit in Alzheimer’s disease. Ann Neurol. 1992 Sep;32(3):371–5.

Response:

Thank you for this important comment. Following the comment, we have revised the whole Introduction and Discussion sections. As pointed out, education is an important factor associated with the cognitive reserve, which reduces the risk of cognitive decline [17]. There are two main difficulties for considering the education level in real clinical data. First, discussing patients’ educational backgrounds has little benefit for them, because they are not modifiable for current patients. Second, patients are often not willing to disclose their education histories. Therefore, in the present study which was a retrospective study using the clinical records, we could not consider education levels as a primary factor. Of note, although we focused on regular physical activity, we are not only interested in its pure beneficial effect. All lifestyle-associated factors interact with each other and work synergically to reduce (or increase) the risk of cognitive decline. For example, the education level has also been associated with regular physical activity [25,26]. It is neither practical nor meaningful to discuss pure contributions of individual lifestyle-associated factors, at least not from a clinical perspective. We focused on regular physical activity as a representative index of lifestyle-associated factors, without excluding influences from other lifestyle-related factors including education (page 7, lines 125-126).

Nonetheless, from an academic perspective, we admit the presence of uncontrolled factors, including education, which may affect the MEG spectral parameters (partly) independently from other lifestyle-associated factors such as physical activity. We cannot rule out the possibility; thus, we have described it as the third limitation (pages 28-29, lines 450-458). We hope that these changes properly addressed your concerns.

Comment 5-9:

Other issue: The last paragraph of the discussion section where the authors discuss why some patients in the active group use functional reserve to improve their current cognitive state [lines 287-305] is highly speculative, as claims are not directly related to or backed up by the authors’ results. Particularly, lines 298-305 present an oversimplification of how electrophysiological neural dynamics are generated and linked to brain disease. Many of these claims need to be toned down.

Response:

Thank you for your thoughtful suggestion. We agree with the concerns and admit that our original Discussion section was not logically written regarding the links to the obtained results (re-reading the Discussion section, we noted some other parts also suffered from similar issues). We have rewritten the Discussion section thoroughly and removed the concept of ‘functional reserve’. A similar concept, ‘cognitive reserve’, was introduced as a potential interpretation of the results, where we explained its link to the MEG parameters used in the study (pages 25-26, lines 416-431). We hope that the reviewer finds the revised manuscript improved and acceptable for publication.

References

1.	Hirata Y, Hoshi H, Kobayashi M, Shibamiya K, Fukasawa K, Ichikawa S, et al. Monitoring the outcomes of non-pharmacological treatments for cognitive impairment using magnetoencephalography: A case series. Clin case reports. 2023 Jan 1;12(1):e8385.

2.	Hoshi H, Hirata Y, Kobayashi M, Sakamoto Y, Fukasawa K, Ichikawa S, et al. Distinctive effects of executive dysfunction and loss of learning/memory abilities on resting-state brain activity. Sci Rep. 2022 Mar 2;12(1):1–10.

3.	Sugishita M, Koshizuka Y, Sudou S, Sugishita K, Hemmi I, Karasawa H, et al. The Validity and Reliability of the Japanese Version of the Mini- Mental State Examination（MMSE-J）with the original procedure of the Attention and Calculation Task（2001）. Japanese J Cogn Neurosci. 2018;20(2):91–110.

4.	Kugo A, Terada S, Ata T, Ido Y, Kado Y, Ishihara T, et al. Japanese version of the Frontal Assessment Battery for dementia. Psychiatry Res. 2007 Sep 30;153(1):69–75.

5.	Nakaaki S, Murata Y, Sato J, Shinagawa Y, Matsui T, Tatsumi H, et al. Reliability and validity of the Japanese version of the Frontal Assessment Battery in patients with the frontal variant of frontotemporal dementia. Psychiatry Clin Neurosci. 2007 Feb;61(1):78–83.

6.	Sakai K, Hosoi Y, Harada Y, Morikawa K, Kato Y. Validity and Reliability of the Japanese Version of the Frontal Assessment Battery in Patients with Stroke. Neurol Int. 2024 Oct 1;16(5):1086–93.

7.	WHO. Risk Reduction of Cognitive Decline and Dementia: WHO Guidelines. Who. 2019. 96 p.

8.	De la Rosa A, Olaso-Gonzalez G, Arc-Chagnaud C, Millan F, Salvador-Pascual A, García-Lucerga C, et al. Physical exercise in the prevention and treatment of Alzheimer’s disease. J Sport Heal Sci. 2020 Sep 1;9(5):394–404.

9.	Pescatello LS, Buchner DM, Jakicic JM, Powell KE, Kraus WE, Bloodgood B, et al. Physical Activity to Prevent and Treat Hypertension: A Systematic Review. Med Sci Sports Exerc. 2019 Jun 1;51(6):1314–23.

10.	Fiscella AJ, Andel R. The association between physical activity, obesity, and cognition in middle-aged and older adults. J Aging Phys Act. 2024 Jun 1;32(3):397–407.

11.	Aune D, Norat T, Leitzmann M, Tonstad S, Vatten LJ. Physical activity and the risk of type 2 diabetes: A systematic review and dose-response meta-analysis. Eur J Epidemiol. 2015 Jul 1;30(7):529–42.

12.	Nakamura A, Cuesta P, Fernández A, Arahata Y, Iwata K, Kuratsubo I, et al. Electromagnetic signatures of the preclinical and prodromal stages of Alzheimer’s disease. Brain. 2018 May 1;141(5):1470–85.

13.	Hoshi H, Hirata Y, Fukasawa K, Kobayashi M, Shigihara Y. Oscillatory characteristics of resting-state magnetoencephalography reflect pathological and symptomatic conditions of cognitive impairment. Front Aging Neurosci. 2024;16:1273738.

14.	Matsumoto T, Hoshi H, Hirata Y, Ichikawa S, Fukasawa K, Gonda T, et al. The association between carotid blood flow and resting-state brain activity in patients with cerebrovascular diseases. Sci Rep. 2021 Jul 27;11(1):1–11.

15.	Johnston PR, McIntosh AR, Meltzer JA. Spectral slowing in chronic stroke reflects abnormalities in both periodic and aperiodic neural dynamics. NeuroImage Clin. 2023 Jan 1;37:103277.

16.	Shekoohishooli F, Chella F, Caulo M, Navarra R, Rapino M, Pizzella V, et al. The impact of gliomas on resting-state oscillatory activity and connectivity: A magnetoencephalography study. Neuroimage: Reports. 2021 Dec 1;1(4):100051.

17.	Livingston G, Huntley J, Liu KY, Costafreda SG, Selbæk G, Alladi S, et al. Dementia prevention, intervention, and care: 2024 report of the Lancet standing Commission. Vol. 404, The Lancet. Lancet; 2024. p. 572–628.

18.	Ducharme-Laliberté G, Mellah S, Boller B, Belleville S. More flexible brain activation underlies cognitive reserve in older adults. Neurobiol Aging. 2022 May 1;113:63–72.

19.	Groot C, Van Loenhoud AC, Barkhof F, Van Berckel BNM, Koene T, Teunissen CC, et al. Differential effects of cognitive reserve and brain reserve on cognition in Alzheimer disease. Neurology. 2018 Jan 9;90(2):e149–56.

20.	Kivimäki M, Walker KA, Pentti J, Nyberg ST, Mars N, Vahtera J, et al. Cognitive stimulation in the workplace, plasma proteins, and risk of dementia: Three analyses of population cohort studies. BMJ. 2021 Aug 19;374:1804.

21.	Esiri MM, Chance SA. Cognitive reserve, cortical plasticity and resistance to Alzheimer’s disease. Alzheimer’s Res Ther. 2012 Mar 1;4(2):1–8.

22.	Niso G, Carrasco S, Gudín M, Maestú F, Del-Pozo F, Pereda E. The Diagnostic Process. NeuroImage Clin. 2015 Dec 29;8:503–15.

23.	World Health Organization, WHO. Risk Reduction of Cognitive Decline and Dementia: WHO Guidelines. Who. Geneva: World Health Organization; 2019. 96 p.

24.	Kim SK, Park M. Effectiveness of person-centered care on people with dementia: a systematic review and meta-analysis. Clin Interv Aging. 2017 Feb 17;12:381–97.

25.	Kari JT, Viinikainen J, Böckerman P, Tammelin TH, Pitkänen N, Lehtimäki T, et al. Education leads to a more physically active lifestyle: Evidence based on Mendelian randomization. Scand J Med Sci Sports. 2020 Jul 1;30(7):1194–204.

26.	Kitano N, Fujii Y, Wada A, Kawakami R, Yoshiba K, Yamaguchi D, et al. Associations of Working From Home Frequency With Accelerometer-Measured Physical Activity and Sedentary Behavior in Japanese White-Collar Workers: A Cross-Sectional Analysis of the Meiji Yasuda LifeStyle Study. J Phys Act Health. 2024 Nov 1;21(11):1150–7.
