# Supplementary material for: Regular physical activity affects brain activities in old individuals: an observational study
Source: PLoS One. 2025 Jul 2;20(7):e0326163. doi: 10.1371/journal.pone.0326163 (PMC12220997; doi:10.1371/journal.pone.0326163)
Supplement: Attachment — Submitted filename: Response-to-Reviewers.docx [file pone.0326163.s005.docx]

Response to reviewers:

**Regular physical activity affects brain activities in old individuals:**

**an observational study**

Short title: Physical activity and brain activity

Keisuke Fukasawa^1, ¶^, Hideyuki Hoshi^2,3, ¶^, Yoko Hirata^4^, Momoko Kobayashi^3^, Keita Shibamiya^3^, Sayuri Ichikawa^1^, Yoshihito Shigihara^2,3*^

^1^Clinical Laboratory, Kumagaya General Hospital, Kumagaya City, Saitama, Japan,

^2^Precision Medicine Centre, Hokuto Hospital, 7-5 Kisen, Inada-cho, Obihiro-shi, Hokkaido, Japan

^3^Precision Medicine Centre, Kumagaya General Hospital, Kumagaya City, Saitama, Japan

^4^Department of Neurosurgery, Kumagaya General Hospital, Kumagaya City, Saitama, Japan

* Corresponding author Yoshihito Shigihara

E-mail: [y-shigihara@hokuto7.or.jp](mailto:y-shigihara@hokuto7.or.jp) (YS)

^¶^These authors contributed equally to this work.

To the editor and reviewers:

Thank you for another opportunity to submit a revised draft of our manuscript titled ‘Regular physical activity affects brain activities in old individuals: an observational study’.

We appreciate the time and effort you have dedicated to providing valuable feedback on our manuscript. We are grateful for your insightful comments that helped us improve the quality of our study. We have incorporated changes to reflect all your suggestions.

Please find our point-by-point responses to all comments and concerns below.

**Role of Funder statemen**t

This study was partially supported by RICOH Co., Ltd. The funders had no role in study design, data collection and analysis, decision to publish, or preparation of the manuscript.

**Response to Editor**

Reviewers generally appreciated the authors' efforts to clarify the methods and improve the coherence of the manuscript. However, there is still substantial ambiguity regarding a crucial part of the methods—specifically, the inclusion criteria and the population studied. One reviewer highlighted that the tests used may not be appropriate for healthy aging subjects. It remains unclear whether a mix of healthy subjects and patients with dementia was studied, and if so, what the rationale was for this selection. Please clearly specify the population studied, including diagnoses, and clarify the reasoning behind the choice of tests. Note that the manuscript may not be accepted if the population selection is not coherent with the study’s aim and relevant methods

**Response:**

Thank you for your efforts in editing our manuscript with the reviewers. We understand that the editor raised two concerns: (1) inclusion criteria and (2) target population of the study.

Regarding the inclusion criteria, we have explicitly described the inclusion and exclusion criteria (page 8, lines 139 — 144). This study was conducted to improve clinical MEG examination and provide information for patients’ treatments (pages 6 — 7, lines 110 — 112; page 29, lines 492 — 493). Thus, we designed the study to have the clinical population as real as possible. Therefore, we included almost all patients who visited our outpatient section of dementia care to seek treatment, regardless of their diagnosis (page 9, lines 147 — 149). Lifestyle modification is effective for almost all individuals (See WHO’s risk reduction of cognitive decline and dementia [1]), and there is little benefit in distinguishing them by their diagnoses. We did not mention their clinical diagnoses in the previous version of the manuscript because they are not directly relevant to the scope of the present study. However, the information regarding the diagnosis would allow readers to grasp the quality of the clinical population of the patients; we have added them in the revised manuscript (pages 8 — 9, lines 144-147).

Regarding the study population, as mentioned above, we included as many patients as possible, regardless of their diagnoses (page 9, lines 147 — 149). Reviewer 1 pointed out that ‘the tests used may not be appropriate for healthy aging subjects.’ However, we consider this point to be inapplicable to the present study for four reasons. First, neither ‘mental function’ nor ‘diagnose’ is within the scope of the present study. We are afraid that Reviewer 1 might misunderstand the subject and purpose of the present study. The subject of the present study is ‘brain activity’ and not ‘mental function.’ We have explicitly described this in the title of the manuscript and in lines 21–22 and 110 – 112. The motivation of the present study is to improve clinical MEG to provide better clinical practice for patients who are afraid of dementia. To achieve this goal, we investigated brain activity measured using MEG to evaluate the influence of regular physical activity, which is a modifiable factor for preventing dementia. Although we used neuropsychological assessments (MMSE, FAB, and ADAS-cog) in the present study, they were merely used to adjust the influence of cognitive impairment (i.e., confounding factors) on brain activity; they were not the core of the study. Second, the selection of neuropsychological assessments (MMSE, FAB, and ADAS-cog) as quantifications of the confounding factors is reasonable for the following reasons. (2-1) A previous study has shown that MMSE and FAB scores influence the MF, IAF, and SSE values [2] (page 6, lines 95 — 96). It is unclear whether the Wechsclar memory scale, Trail making tests, and Barthel index influence MEG spectral parameters, although they are likely to do so. (2-2) We intend to use the present results in clinical practice. We should select neurophysiological assessments that are frequently used in the outpatient section for dementia care. We selected the three assessments routinely used in our hospital (Page 11, lines 188 — 191). They are also widely used to screen patients to distinguish individuals who are at risk from healthy individuals. This indicates that the assessments are applicable to both healthy individuals and patients with cognitive impairment. Third, the study protocol used in the present study was also used in our previous studies, which included both healthy individuals and patients with cognitive impairment and dementia [2,3], which supported the validity of the study. Fourth, healthy conditions and cognitive impairment are a continuum [4]. There was no gap between healthy individuals and patients with cognitive impairment. It is unreasonable to claim that the three assessments cannot be used for healthy individuals. Although we appreciate Reviewer 1’s detailed reading and careful evaluation, we consider that the comment would be beside the point.

We believe that our study setting is the best (available) approach, and the present study contributes to the happiness of future patients.

Response to Reviewer 1 Comments

The manuscript described the daily activities affecting the mental function in aged.

There are several problems, and among them I wold like to point out the psychological examinations; adopted tests are for the medical diagnostic test for dementia. It is necessary to measure the normal persons maintaining the brain functions, such as Wechsclar memory scale for memory, Trail making tests for attention, Barthel index for ADL and so on. The psychological examination for people of good normal life could not be evaluated by medical psychological examinations such as muse, or others.

In order to evaluate the brain integrity, it is difficult to do by MEG. In a reverse sense, it is expected that the aged brain have a MEG or EEG function on a particular pattern or not.

In my impression, the design of the study is not good for the intended purpose.

**Response:**

We appreciate the reviewer’s detailed reading and careful evaluation. We are afraid that the reviewer might misunderstand the subject and purpose of the present study. Although they commented that ‘The manuscript described the daily activities affecting the mental function in aged’, mental function is not our main interest. We are interested in the relationship between regular physical activity and brain activity, as described in the title of this manuscript.

Furthermore, we would like to remind that the present study did not focus on the distinction between diagnostic groups (e.g., healthy controls vs. patients with dementia). The motivation for the present study was to improve clinical MEG examination to provide better practice for patients who are afraid of dementia. The retrospective study included a dataset of all eligible patients who visited the outpatient section of dementia to replicate the real clinical population, while their diagnostic and pathological backgrounds were not considered as inclusion/exclusion criteria. Lifestyle modification is effective for everyone, regardless of their diagnosis or clinical state, not only for primary prevention of cognitive impairment but also for secondary and tertiary prevention (See WHO’s risk reduction of cognitive decline and dementia [1]). Thus, it is essential to include all individuals who visited our hospital to seek medical care, regardless of their cognitive state. Therefore, they did not need to be screened or categorised strictly using detailed examinations for the purpose of the present study. To be explicit about the patients’ clinical backgrounds and allow readers to grasp the quality of the clinical population of the patients, we have added these information in the revised manuscript (pages 8 — 9, lines 144 — 147).

Essentially, healthy conditions and cognitive impairment are a continuum [4]. There is no gap between healthy individuals and patients with cognitive impairment. All individuals, especially older individuals, are at risk of dementia; there are no perfectly healthy individuals. Therefore, the claim that the three assessments (MMSE, FAB, and ADAS) cannot be used for healthy individuals in clinical practice is unreasonable. This is supported by the figures in our previous studies [2,3][Figure 2 in Hoshi et al. (2022) [2] and Figure 4 in Hoshi et al. (2023) [3]]. These studies included healthy individuals and patients with cognitive impairment and dementia. They used a strategy similar to the present one and showed that MMSE and FAB can be applied to both healthy individuals and patients with cognitive impairment and dementia.

We used neuropsychological assessments, such as the MMSE, to control for the influence of cognitive level on brain activity. Therefore, ‘mental function’ was a confounding factor in this study. If the main subject of the present study was associated with ‘mental function’, we would agree with the reviewer that the function should be assessed more extensively. However, it is not. We considered the choice of using the three assessments as quantifications of the confounding factors reasonable for the following reasons. First, a previous study has shown that MMSE and FAB scores influence MF, IAF, and SSE values [2] (page 6, lines 95 — 96). It is unclear whether the Wechsclar memory scale, Trail making tests, and Barthel index influence MEG spectral parameters, although they are likely to do so. Second, we routinely use the three neuropsychological assessments in our outpatient department for dementia treatment. We also use other neuropsychological assessments, but not routinely. The motivation of the present study is to improve cognitive treatment for cognitive impairment and dementia; thus, the frequently used assessments were naturally the focus of the study.

As a summary, if the main subject of the present study was associated with ‘mental function’, we would agree with the reviewer that the function should be assessed more extensively. However, it is not. Therefore, we believe that the present study design is reasonable and practical for addressing the present subject.

Response to Reviewer 2 Comments

l. 329 “ANCOVA and SOC” The title of the section does not give any information about the topic.

I think a better idea is from your text below "Effect of groups on MEG spectral parameters".

**Response:**

Thank you very much for your constructive suggestions. Following the comment, we have changed the title of the subsection as suggested (page 22, line 359).

**Response:**

l. 357 Table 2. Results of Analysis of Covariance. I already pointed out this inaccuracy in the previous review (see comment 2-15). It is better to give the table title from your text “Assessing the effects of regular physical activity on MEG spectral parameters”. In your reply to me, you wrote that you had corrected this inaccuracy, but apparently you forgot to do so in the text.

Thank you for your suggestion. Following the comment, we have modified the title of Table 2 as suggested (page 23, line 386). We apologise that our modification in the previous round of review was not satisfactory to the reviewer.

**Response to Reviewer 5 Comments**

Minor points

1) Line 56. Typo. Its process -> This process

2) Line 62. Typo. Influence on pathological conditions -> influence pathological conditions

3) Line 85-86. Which are sensitive to the cognitive decline -> which are sensitive to cognitive decline

4) Figure 1. Bottom left corner. Week association -> Weak association

5) Line 100. and considered drivers of cognitive impairments -> and are considered drivers of cognitive impairments

6) Line 373. These factors interfere each other -> these factors Interfere with each other

7) Lines 375 and 378 as the direct/indirect pathways -> as direct/indirect pathways

8) Line 381. From a clinical perspective it is not essential whether… -> From a clinical perspective, it is not clear whether…

9) Line 385. This study showed that MEG spectral parameters were enhanced by regular physical activity ----- Enhanced might be an inappropriate word choice here. Consider using less causal language.

10) Line 388. Contain -> Reflect

**Response:**

We appreciate the reviewer for the detailed reading and thank you very much for finding typos and making suggestions. Following your comments, we have modified/corrected them. Regarding comment 9), we used ‘associated’ instead of ‘enhanced’ to describe the indirect and non-causal relationships between the MEG spectral parameters and regular physical activity (page 26, line 418).

11) Line 425. Theoretically, an increase in high-frequency oscillatory activity corresponds to high MF, IAF, and SSE values. ----- This is highly speculative, as spectral parameters are jointly modulated by a myriad of neurophysiological and neuromodulatory phenomena, rather than oscillatory power within a single frequency band. I would suggest the authors to either remove this claim or substantiate it with empirical evidence.

**Response:**

Thank you for your constructive comments. Following this comment, we removed the sentence (page 28, line 455).

Final comments

I would like to thank the authors for taking the time to implement the suggested corrections and submit a much-improved version of their original work. I enjoyed reading this new version and found their line of argumentation much clearer. The description of data analysis and statistical procedures is much clearer and logically responds to their research question. I also appreciate the inclusion of MEG data acquisition and preprocessing procedures. I am still somewhat confused as to why the authors chose to use SOC to quantify the effect of physical activity on predicting MEG spectral parameters, rather than more conventional methods such as mediation analyses. However, I do appreciate the mathematical and statistical explanation provided by the authors, which allowed me to understand their approach better than in the original submission.

**Response:**

Thank you for your kind message. Regarding the employment of SOC, we used the strategy to quantify the effect of regular physical activity using the language (i.e., unit) of neuropsychological assessment but not that of neurological parameters (i.e., MEG spectral parameters). For clinicians involved in the care of patients with cognitive decline, it would be difficult to comprehend the significance of the results, such as ‘the patients with regular physical exercise showed 2.0 point higher MF than those without’, because electrophysiological examinations are not in the mainstream of the clinical practice of cognitive decline. Therefore, we attempted to convert it using the SOC and described that ‘the patients with regular physical exercise showed higher MF than those without, which is equivalent to 4.1 points in MMSE-J’, which should be much more comprehensible to clinicians.

We agree with the reviewer that the use of mediation analysis would be interesting to examine the model described in Figure 1; we might be able to assess the mediative role of neurological parameters (i.e., MEG spectral parameters) between regular physical activities and cognitive state (i.e., neuropsychological assessments). The conversion of the unit between MEG spectral parameters and neuropsychological scores would also be possible using the coefficients of mediation analysis. However, we believe that detailed causal relationships should be assessed using longitudinal data instead of the retrospective cross-sectional data used in the present study. To avoid overinterpretation of the cross-sectional dataset, we did not directly assess the mediative model and limited ourselves to examining the simple ANCOVA model. We are now planning to collect longitudinal data to assess the model in Figure 1, where mediative analysis would be suitable. We hope that the reviewer understands our strategy.

The only thing I am missing from this new version is a more careful explanation of what the different spectral parameters included in this study measure, neurophysiologically speaking (i.e., changes in excitation/inhibition ratios, changes in neuromodulator release, power ratios between different frequency bands, etc.). This would help the reader make a smoother connection between physical activity, brain physiology, and cognition.

**Response:**

We thank the reviewer for encouraging us to describe this. We have added this information to the Methods section (pages 14 — 15, lines 245 — 269). We avoided detailed writing in the previous version because it may sound too detailed and out of interest to some readers of the journal, although we totally agree with the reviewer that these descriptions are important. We hope that the revised version responds well to the reviewers’ suggestions.

**References**

1.	World Health Organization, WHO. Risk Reduction of Cognitive Decline and Dementia: WHO Guidelines. Who. Geneva: World Health Organization; 2019. 96 p.

2.	Hoshi H, Hirata Y, Kobayashi M, Sakamoto Y, Fukasawa K, Ichikawa S, et al. Distinctive effects of executive dysfunction and loss of learning/memory abilities on resting-state brain activity. Sci Rep. 2022 Mar 2;12(1):1–10.

3.	Hoshi H, Kobayashi M, Hirata Y, Fukasawa K, Ichikawa S, Shigihara Y. Decreased beta-band activity in left supramarginal gyrus reflects cognitive decline: Evidence from a large clinical dataset in patients with dementia. Hum Brain Mapp. 2023 Dec 1;44(17):6214–26.

4.	Walters GD. Dementia: Continuum or Distinct Entity? Psychol Aging. 2010 Sep;25(3):534.
