# Supplementary material for: Regular physical activity affects brain activities in old individuals: an observational study
Source: PLoS One. 2025 Jul 2;20(7):e0326163. doi: 10.1371/journal.pone.0326163 (PMC12220997; doi:10.1371/journal.pone.0326163)
Supplement: Attachment — Submitted filename: Response-to-Reviewers_auresp_3.docx [file pone.0326163.s006.docx]

Response to reviewers (for Appeal):

**Regular physical activity affects brain activities in old individuals:**

**an observational study**

Short title: Physical activity and brain activity

ID: PONE-D-24-41991R2

Keisuke Fukasawa1, ¶, Hideyuki Hoshi2,3, ¶, Yoko Hirata4, Momoko Kobayashi3, Keita Shibamiya3, Sayuri Ichikawa1, Yoshihito Shigihara2,3*

1Clinical Laboratory, Kumagaya General Hospital, Kumagaya City, Saitama, Japan,

2Precision Medicine Centre, Hokuto Hospital, 7-5 Kisen, Inada-cho, Obihiro-shi, Hokkaido, Japan

3Precision Medicine Centre, Kumagaya General Hospital, Kumagaya City, Saitama, Japan

4Department of Neurosurgery, Kumagaya General Hospital, Kumagaya City, Saitama, Japan

* Corresponding author Yoshihito Shigihara

E-mail: y-shigihara@hokuto7.or.jp (YS)

¶These authors contributed equally to this work.

Comment 1.

The study suffers from significant sampling bias. The studied sample is not representative of a specific population (e.g. healthy elderly subjects, early AD, etc), but simply reflects the people that seek consulting at the involved Medical Centre. This is not a meaningful population and can’t be easily generalized.

Response 1:

While we appreciate the careful review of and detailed feedback regarding our manuscript, we feel that the word ‘bias’ was used incorrectly here. As we explicitly described in the manuscript (lines from 110–112) and also clarified in our responses to the reviewers, the goal of the present study was to develop a clinical tool to facilitate the assessment of lifestyle-associated factors in clinical environments. We strongly believe that, to achieve this purpose, the study cohort **must** comprise patients who seek lifestyle intervention (lines from 147–149). Therefore, we have used the presented dataset which included patients who visited a hospital for that purpose. While the editor stated that the effects should be examined in a group of patients with/without specific diagnostic (pathological) backgrounds, we believe that such a selection would actually bias our sample. In general, we would be happy to perform additional analyses of interest within subgroups of our cohort; however, considering the original goal of our study, we do not see how such additional analyses would fit within the scope of the current manuscript. We would appreciate if the editor could be clearer regarding the reason for recommending using datasets from patients subgroups to address the purpose of the study.

Comment 2.

Moreover, only in the last revision authors listed the outcome of the diagnostic path, stating that among the subjects “32 were diagnosed with healthy ageing, 60 with mild cognitive impairment, and 220 with dementia”. The sample was treated as a whole, irrespectively of health condition, and the statistical analysis contrasted active vs non-active, with no covariate related to diagnosis. Instead, neuropsychological assessments were used as covariates, and they may be inappropriate across the involved sub-populations. Irrespectively of the covariates, this approach obviously prevented the study of interactions activity x health condition. It may well be that the reported effect is dominated by one sub-group.

Response 2:

As we previously responded to the reviewers (lines from 47–48, 66–68, 115–117, and 149–151) in the second round, clinical diagnoses were not within the scope of the present study. The goal of the present study was to develop a clinical tool to assess lifestyle-associated factors in clinical environments. In real-life diagnostic processes in the clinical practice, diagnoses follow examinations; i.e., at the time of clinical examinations, including MEG, there are no established diagnoses. Clinicians make diagnoses based on medical interviews and clinical examinations. The results of the MEG examinations, including the quantification of the influence of regular physical activity on brain activity, are considered during the diagnosing process. Therefore, for evaluating the influence of regular physical activity on MEG spectral parameters, diagnosis information is not available. Consequently, we believe that the editor’s suggested model including diagnostic information would be unsuitable in the clinical practice. In contrast, neuropsychological assessment scores are usually available at the time of MEG examinations, because these assessments may happen on the same day of the MEG scan, which always precedes the diagnosing day. Keeping the purpose of the present study and the actual clinical environment in mind, we hope that the editor will agree with the suitability of our presented approach.

Comment 3

Finally, the manuscript, including the title, refers generally to “old individuals”, and states that findings can be used in patients with cognitive impairment. All of these are undue generalizations, if the sampled population does not match the claims.

Response 3:

We strongly disagree with this comment. To generalize the results to “old individuals”, the studied population must include old individuals from all backgrounds without any further prerequisites. The goal of the present study was to develop a clinical tool to assess lifestyle-associated factors in clinical environments (lines 47–48, 66–68, 115–117, and 149–151 in the response letter of the second round). The study cohort comprised older individuals who visited a hospital to seek medical care, which genuinely represents “old individuals”. We would appreciate if the editor could be more explicit as to why they believe that the study population did not match “old individuals” and explain which populations would satisfies their expectation.
